# Simulation Enhances Resident Preparedness Using Skin Cell Suspension Autograft

**DOI:** 10.3390/ebj7020031

**Published:** 2026-05-21

**Authors:** Joshua P. Kronenfeld, Louis R. Pizano, Ray I. Gonzalez, Joyce I. Kaufman, Shevonne Satahoo, Carl I. Schulman

**Affiliations:** Division of Trauma, Surgical Critical Care & Burns, Daughtry Family Department of Surgery, University of Miami Miller School of Medicine and Jackson Memorial Hospital Ryder Trauma Center, Miami, FL 33136, USA; j.kronenfeld@umiami.edu (J.P.K.); lpizano@miami.edu (L.R.P.);

**Keywords:** burns, surgery, simulation, training, residency

## Abstract

Objective: Surgical simulation has been shown to improve efficiency, performance, and time to mastery for complicated procedures, but simulation training is not always considered when introducing new devices or products. As part of a performance improvement project, we sought to design and evaluate simulation training for the skin cell suspension autograft (SCSA) with surgery residents during their Burn rotation. Methods: Residents were asked to read instructional materials and watch training videos before coming into the simulation lab for the training session supervised by a Burn surgeon. A qualitative survey was designed and administered after completion of the rotation. Results: Twelve residents have completed the training thus far. Their feedback from the training session was rated on a five-point Likert scale and indicated that the simulation activity was an appropriate length (4.6/5.0), was thorough (4.8/5.0), and led to more confidence (4.4/5.0) and less apprehension (4.4/5.0) when performing the procedure on live patients. This was followed by their use of the product in the operating room with complete success. Conclusions: The novel SCSA training shows great promise for improving the confidence and performance of surgical residents. This could allow for a shorter time for residents to become independent in its use, thereby allowing for increased operative efficiency with the opportunity to significantly improve trainee expertise.

## 1. Introduction

Surgical simulation has been shown to improve efficacy and performance, and shorten time to mastery of surgical skills [1]. Historically, surgeons would need to study independently or observe other surgeons in order to learn a new surgical technique or procedure [2]. In fact, the giants of modern surgery, such as Theodor Billroth, taught new techniques to their students and trainees by performing operations with large audiences in a grand operating theater [3]. For years, surgeons would use surgical atlases or published manuscripts to detail new procedures or techniques. After only reading about them, surgeons would then attempt these, often complex, procedures on human patients. In more recent memory, surgeons have shared videos of their operations with colleagues at conferences or through online platforms [4]. This has expanded the influence of individual surgeons and allowed their techniques to reach a wider audience.

Although these aforementioned training modalities may have sufficed when few alternatives were available, studies have demonstrated that effective simulation trainings can provide added confidence to surgeons and improved outcomes for patients [1]. Initially utilized for extremely complex surgical operations, such as congenital cardiothoracic procedures, the scope of surgical simulation uses has drastically expanded [5,6]. In fact, surgical simulation has been increasingly utilized in subspecialties such as surgical endoscopy, gynecologic surgery, urologic surgery, and many other disciplines [6,7,8,9]. The use of simulation requires the development of a curriculum by a surgical expert. The trainees and students can then follow this curriculum while performing the described procedures on the selected media [10]. The utilization of these techniques can provide confidence to trainees and students, allowing them to perform a procedure with improved technical skill once operating on human patients.

While previous studies have demonstrated the efficacy of surgical simulation on accelerating mastery of skills, limited information is available regarding these techniques in the Burn surgery literature [8]. In this study, surgical trainees were exposed to an extensive training program on a novel burn surgical procedure, skin cell suspension autograft (SCSA). This surgical procedure requires a precise, multi-step process that must be performed exactly as directed or the procedure will not be successful. Deviation from the exact procedure will lead to loss of skin that was harvested as well as loss of the cost of equipment to produce the skin suspension. Since this was a novel procedure at our medical center, attending surgeons requested this training session to better equip trainees to successfully perform this procedure without unnecessarily damaging harvested skin. The trainees then practiced these techniques using a simulation exercise that utilized a tissue-based model. The aims of this performance improvement (PI) project were to understand the effectiveness of this simulation modality in preparing trainees to perform it autonomously and to identify any areas for improvement for future trainees. We hypothesized that trainees would be very receptive to this form of education and have improved confidence in their abilities upon implementing these surgical techniques on real patients.

## 2. Materials and Methods

### 2.1. Study Design

This study was designed as a descriptive post-intervention survey aiming to achieve preliminary data for this novel curriculum. The study was designed to be hypothesis generating and to demonstrate the feasibility of developing such a curriculum. Additionally, the study does not contain a comparative arm as the participants in this study were not surveyed pre-intervention, so it is purely a descriptive study as noted above.

### 2.2. Participant Selection

General surgery and plastic surgery trainees, rotating on the Burn surgery service, were eligible for this training if they were participating in the rotation during their third clinical year of training between 2022 and 2024, starting in June of 2022. At the conclusion of their rotation, an anonymous voluntary survey was administered. Survey questions focused on the surgical simulation exercise to learn about SCSA, a spray-on skin graft solution from harvested skin. The University of Miami Institutional Review Board determined that review of our performance improvement project was not human subject research. Although this was a performance improvement project, all participants still consented to the study and survey before participating.

### 2.3. Design of Curriculum

**Figure 1:**
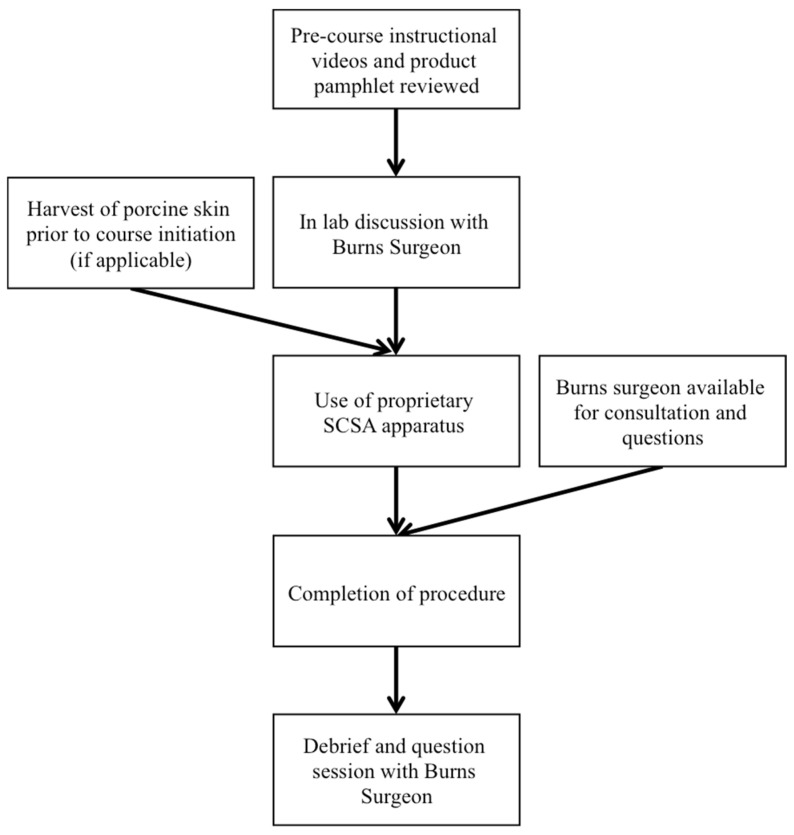
Workflow detailing the training sessions.

At the start of their third-year Burn rotation, which runs for six to eight weeks, residents were given a simulation activity after preparing for the session by reading a product pamphlet and reviewing an instructional video regarding the SCSA product (Figure 1). The simulation session lasted from one to two hours and was supervised by a Burn surgery attending, who remained immediately available for questions or clarifications. The session utilized the same instruments, chemical reagents, and proprietary SCSA apparatus that would be used in the operating room with a live patient. The kits that were utilized for the training sessions were expired kits provided by the company at no cost to the institution. Initially, instead of harvesting live tissue, sections of standard computer paper were used to simulate human skin. After the first two resident trainings, however, porcine skin was available and used for all subsequent trainings, including the other ten trainees included in this study. While there was variability from the first two residents to the subsequent ten trainees in the medium used to simulate human skin, all other methodologies remained consistent in this study. The porcine skin was harvested, using a pneumatic dermatome, from animals that had been previously used for other resident training programs. All ethical standards required by the institution were adhered to, and all personnel harvesting the tissues had completed required research training modules for human and animal research. Upon completion of this simulation exercise, residents then participated in operations on live human patients as part of their Burn rotation that utilized the practiced techniques. The number of cases each resident recorded was variable and depended on patient necessity during the resident’s six- to eight-week rotations. At the completion of the rotation, residents were surveyed regarding their experience with the simulation session (Appendix A).

### 2.4. Variables

Demographics were not obtained, in order to maintain anonymity in a residency program with less than ten trainees per year. The primary endpoints were survey responses, which provided information on whether the simulation activity was an appropriate length, whether the simulation activity was thorough, if residents felt more confident in performing the procedure after the simulation, if trainees were less apprehensive about using the SCSA technique in the operating room, if the simulation improved the ability to use the product in the operating room, and if residents became independent in the operating room quicker as a result of the simulation. Residents also detailed the number of times they utilized the techniques in the operating room on live tissue, after completing the simulation session.

### 2.5. Statistical Analysis

Descriptive statistics, including mean and standard deviation (SD), were calculated for the number of times these techniques were utilized in the operating room on live tissue. A five-point Likert scale was used to assess the other variables including: whether the simulation activity was an appropriate length, whether the simulation activity was thorough, if residents felt more confident in performing the procedure after the simulation, if trainees were less apprehensive about using the SCSA technique in the operating room, if the simulation improved the ability to use the product in the operating room, and if residents became independent in the operating room quicker as a result of the simulation. The Likert scale included the following responses: strongly disagree (1 point), disagree (2 points), neutral (3 points), agree (4 points), and strongly agree (5 points). Mean and SD were calculated for these responses as well. Statistical analysis and figure generation were performed using Microsoft Excel 2011 (Microsoft, Redmond, WA, USA).

## 3. Results

### Survey Results

**Figure 2:**
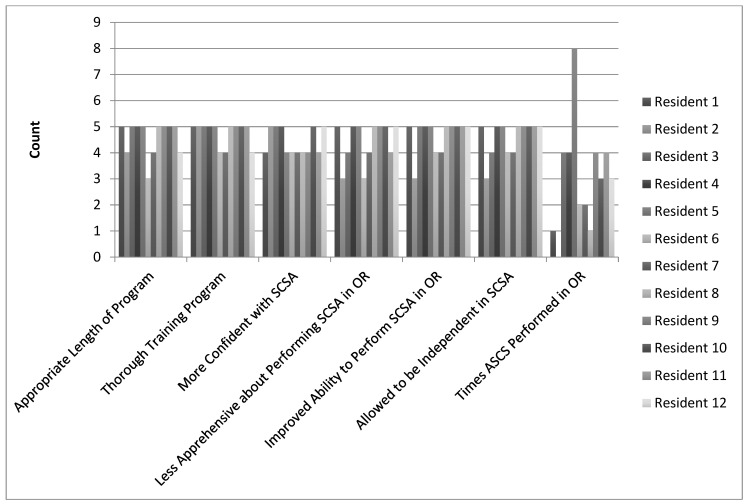
Responses to survey questions, organized by resident.

Over a two-year period, 12 residents completed the simulation training, read the product pamphlet, and reviewed the instructional video prior to their Burn rotation, and the survey response rate was 100% with all residents meeting inclusion criteria. Residents indicated that training sessions were an appropriate length (4.6 ± 0.7), thorough (4.8 ± 0.5), made them more confident in performing the procedure (4.4 ± 0.5), made them less apprehensive about performing the procedure in the operating room (4.4 ± 0.8), improved their ability to perform the procedure in the operating room (4.7 ± 0.7), and allowed them to be independent when performing the procedure in the operating room (4.6 ± 0.7) (Table 1, Figure 2). Residents performed the procedure an average of 3.0 (SD 2.1) times during their Burns surgery rotation.

## 4. Discussion

Simulation is a novel technique that has been utilized to train individuals on a particular task [1]. This study details the implementation of a simulation-training program that was implemented on the Burn surgery rotation at an American Burn Association (ABA)-verified Burn Center. While historically residents would learn new techniques by shadowing a trained surgeon or reading about procedures in a surgical atlas or textbook, newer techniques, such as simulation-based learning, have demonstrated improved outcomes [2]. Through surgical simulation, residents demonstrate a more rapid mastery of a particular skill, and they are more confident and competent when performing these skills on live patients [5]. This PI project has demonstrated similar results, and residents have indicated that they were more confident, had less apprehension, and had improved ability to perform SCSA after completing the training program. This performance improvement study may help to design further studies to investigate the benefits of simulation activities. Additionally, the significant increase in trainee comfort with performing this procedure may suggest that other complex procedures should be first learned in a simulated setting before performing them on patients.

When designing a simulation program, it is important that information be concise but thorough. The military has designed numerous simulation exercises to train soldiers to complete certain tasks or respond to a particular situation [11]. One such example that has been utilized for many years is the use of flight simulation to train military pilots. Prior to flying an aircraft, trainees are required to complete a certain number of hours on simulation exercises, and they must demonstrate competency in specific tasks [11,12]. Aviation specialists have studied the attributes of an effective training model when designing these programs [13]. Programs must be thorough and have an appropriate length in order to inspire confidence and achieve competence in an exercise [14]. In this study, surgical trainees identified that both of these attributes were present in the SCSA training sessions. While there are other qualities of an effective training exercise, such as repetition, team training, ability to transfer to practice, and skills maintenance, this study is only a preliminary investigation. Further studies may investigate the integration of these skills within our program as additional residents are trained.

A potential drawback from simulation-based education is that each learner has different methods in which they best retain information and skills. Some residents may be able to master a skill after only one attempt at practicing the task [15]. Other trainees may require repetitive simulations in order to be competent in a particular surgical skill. Additionally, some learners are able to absorb information adequately in an individualized session, while others report improved learning if they work alongside others in a small group setting. In the past few years, there has been a shift in medical education to highlight problem-based learning in a group setting to address this concern [16,17]. Since the operating room represents a group setting where multiple providers (attendings, residents, students, nurses, etc.) must work together to achieve a common goal, integrating the entire team in the simulation exercises may be of benefit in future investigations.

An additional concern regarding surgical simulation is the cost related to implementing these programs. In order to conduct these simulation exercises, appropriate materials must be purchased or obtained, and a simulation center must be available to administer these sessions [10]. The materials must accurately resemble the targeted procedure in order for the training to be successful. In this study, porcine skin was used as a surrogate for human skin when performing the SCSA procedure. While this may have been an adequate substitute for this training model, other procedures may not have such a readily available and cheap surrogate for the technique being demonstrated. This can lead to high costs or ineffective simulation trainings. Additionally, the attending surgeon must take time away from clinical duties, which may influence salaries in a relative value unit model [18]. This may be ameliorated at teaching institutions where faculty members have specific time dedicated to resident and student education, but it may be a challenge in private hospital systems.

This study has certain limitations that should be addressed. There is a limited sample size as only 12 residents were included in this study. Surgical residency training programs are often small, with six to eight general surgery residents graduating each year at the institution that conducted this study. The length of this study was less than two years, so the number of residents who rotated through the Burn service was limited. Additionally, due to scheduling reasons and lab availability, some residents were not able to be trained prior to their first use of the SCSA product in the operating room, so they were not included in this training program or included in this study. An additional limitation of this study is recall bias. The surveys were completed at the end of the six- to eight-week Burn rotation, but the training session was completed within the first two weeks of starting on service. Residents may not have fully recalled the training session or its effects on their performance and confidence in the operating room when performing the SCSA procedure. A coercive bias may also have existed in resident completion of the survey as their responses would be reviewed by the supervising attending physicians who administered the training modules. Yet another limitation to this study is that no pre-training survey was conducted to identify the resident’s baseline understanding of the SCSA as well as their comfort in using the system prior to undergoing the training. Finally, this study evaluated only the trainee’s response to a survey following the training and did not include an objective structured assessment of technical skills to identify if the techniques and principles learned from the training were appropriately translated to the operating room. Continued efforts are needed to identify the long-term implications of this simulation-based training exercise.

## 5. Conclusions

In this subjective, survey-based study, the SCSA simulation-training program has demonstrated early promise in effectively preparing residents to perform the procedure in the operating room. Additionally, the training program has demonstrated that simulation exercises may be helpful when training surgical residents to perform complex Burn surgery procedures. While additional studies may be required to identify long-term implications for this program, this study demonstrates that future efforts are worthwhile as we continue to explore the benefits of surgical simulation for trainees. If significant improvements in preparedness for operative procedures are noted on repeat studies, there may be a shift in training modalities utilized by surgical training programs.

## Figures and Tables

**Table 1:** Survey results from 12 surgical residents at the completion of the Burn surgery rotation.

Survey Question	Mean ± SD
Appropriate Length of Program	4.6 ± 0.7
Thorough Training Program	4.8 ± 0.5
More Confident with SCSA	4.4 ± 0.5
Less Apprehensive about Performing SCSA in OR	4.4 ± 0.8
Improved Ability to Perform SCSA in OR	4.7 ± 0.7
Allowed to be Independent in SCSA	4.6 ± 0.7
Times SCSA Performed in OR	3.0 ± 2.1

SCSA: Skin Cell Suspension Autograft; OR: Operating Room; SD: Standard Deviation.

**Figure A1:**
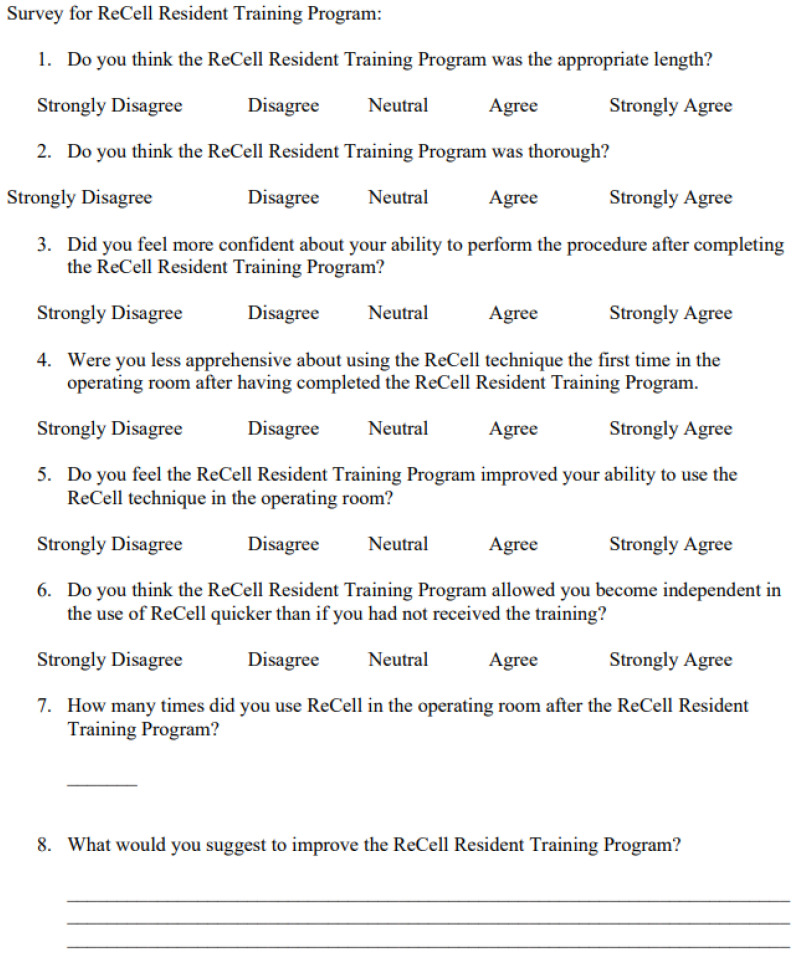
Survey for Training Program.

## Data Availability

The data presented in this study are available on request from the corresponding author due to privacy.

## Abbreviations

The following abbreviations are used in this manuscript:

SCSA	Skin cell suspension autograft
PI	Performance improvement
SD	Standard deviation
ABA	American Burn Association
